# Cross-cultural adaptation and validation of the Healthy Work Environment Assessment Tool in Brazilian culture

**DOI:** 10.1590/0034-7167-2023-0505

**Published:** 2024-09-20

**Authors:** Renata Cristina Gasparino, Sharlla Milênia Nogueira da Silva, Letícia Bianchini de Barros Minatogawa, Olga Maria Pimenta Lopes Ribeiro, Andrea Bernardes

**Affiliations:** I Universidade de São Paulo. Ribeirão Preto, São Paulo, Brazil; II Universidade Estadual de Campinas. Campinas, São Paulo, Brazil; III Escola Superior de Enfermagem do Porto. Porto, Portugal

**Keywords:** Translating, Cross-Cultural Comparison, Validation Study, Health Facility Environment, Working Conditions, Traducción, Comparación Transcultural, Estudio de Validación, Ambiente de Instituciones de Salud, Condiciones de Trabajo

## Abstract

**Objectives:**

to adapt and validate the content of the Healthy Work Environment Assessment Tool for Brazilian culture, and evaluate the practical aspects of its application.

**Methods:**

methodological study that followed six stages: translation; synthesis; back translation; content validation by a group of experts, pre-testing and approval of the process by the author of the original instrument.

**Results:**

the first three stages were carried out by contracted companies. In the committee, two items and the title of a subscale were evaluated in a second round, when consensus was reached among experts. In the pre-test, more than 93% of professionals agreed that the tool was easy to understand. The average completion time was 8.53 minutes. The American Association of Critical-Care Nurses authorized publication of the results.

**Conclusions:**

the adaptation of the tool to Brazilian culture was completed following the adopted framework. In addition to the evidence of content validity, the tool appears promising for managerial use.

## RESUMEN

**Objetivos:**

adaptar y validar el contenido de la *Healthy Work Environment Assessment Tool* a la cultura brasileña y estimar los aspectos prácticos de su aplicación.

**Métodos:**

se trata de un estudio metodológico realizado en seis etapas: traducción; síntesis; retro-traducción; validación del contenido por un grupo de expertos, pre-test y aprobación del proceso por el autor del instrumento original.

**Resultados:**

las tres primeras etapas fueron llevadas a cabo por empresas contratadas. En el comité, dos ítems y el título de una sub-escala se evaluaron en una segunda ronda, cuando se alcanzó un consenso entre los expertos. En la prueba previa o pre-test, más del 93% de los profesionales coincidieron en que la herramienta era de fácil comprensión. El tiempo medio de cumplimentación fue de 8,53 minutos. La Asociación Americana de Enfermeras de Cuidados Críticos autorizó la publicación de los resultados.

**Conclusiones:**

la adaptación de la herramienta a la cultura brasileña se completó siguiendo el marco adoptado. Además de las pruebas de validez de contenido, la herramienta es ideal para el proceso de gestión.

## INTRODUCTION

Currently, the relationship between the characteristics of the work environment and the health of professionals has been assessed, as there are several factors that can contribute to psychosocial and economic implications, not only at the individual level, but also at the family, organizational and social level. Thus, the work environment is seen as a crucial “arena” for promoting the health of the population, which cannot be minimized^(1)^.

In this sense, the World Health Organization (WHO) defined a healthy work environment as a place in which “workers and managers collaborate to use a process of continuous improvement in the protection and promotion of safety, health and well-being of all workers and for the sustainability of the work environment”^(2)^. This construct is considered fundamental in the health area, especially to guarantee health care for those involved, satisfaction and retention of professionals, patient safety, in addition to helping to maintain the financial viability of organizations^(3-4)^.

The theme of a healthy work environment was the focus of the 11^th^ Institutional Seminar of the Federal Nursing Council (Cofen), held in 2021. At the event, it was considered that Brazil is experiencing a second pandemic as a result of Covid-19, now related to mental health^(5)^. This assessment was reinforced by the report from the International Labor Organization (ILO), which highlights that although work acts as a potential protective factor for mental health by providing structure, social interactions, a sense of collective effort and purpose, it can also contribute to a process of psychological illness. As a result, it is estimated that 12 billion working days are lost annually due to depression and anxiety^(6)^.

To develop a healthy work environment, the WHO indicates suggests that an initial diagnosis should be carried out, so that managers can later develop a process of continuous improvement. The initial stages of this process consist of the mobilization of workers and employers and work team meetings to promote changes in the environment^(2)^. Therefore, it is important to highlight that the use of tools with evidence of validity and reliability are essential to evaluate certain patterns present in environments and guide transformations in the places where the work is carried out.

In view of this, the American Association of Critical-Care Nurses (AACN) developed the Healthy Work Environment Assessment Tool (HWEAT), an instrument that aims to serve as a facilitator in identifying areas of improvement in the work environment in the healthcare sector. Furthermore, it stands out for its ability to be applied in different units and consider the perception of the entire multidisciplinary team^(7)^, which will enhance the possibility of generating changes that positively impact the qualification of work environments and, simultaneously, the health and well-being of the entire team. Furthermore, HWEAT was considered by authors who conducted a systematic review as one of the three most used instruments to assess the characteristics of the work environment^(3)^.

This tool had its items divided into six standard dimensions: communication, true collaboration, effectiveness in decision making, appropriate team, significant recognition and authentic leadership. It is worth highlighting that standards lead to continuous quality improvement^(7)^. Furthermore, authors showed that a work environment with favorable characteristics is a central impact factor for obtaining satisfactory results related to nurses, patients and institutions^(3)^. HWEAT had its reliability tested with the multidisciplinary team and achieved Cronbach’s alpha values that varied between 0.77 - 0.81^(7)^.

Considering that Brazilian literature does not yet have an instrument that assesses the characteristics of a healthy work environment from the perspective of the multidisciplinary team and that there is evidence that points to the need to carry out assessments of work environments in the health sector, he believes It is believed that the availability of HWEAT for Brazilian culture could contribute to the identification of areas for improvement and the development of strategies that could contribute to the promotion of healthier environments and, consequently, to achieving more favorable results for professionals, patients and institutions.

## OBJECTIVES

To adapt and validate the content of the Health Work Environment Assessment Tool for Brazilian culture and evaluate the practical aspects of its application.

## METHODS

### Ethical aspects

The author of the original instrument granted consent for the process of cross-cultural adaptation and validation of HWEAT for the Brazilian context. Furthermore, the study was approved by those responsible for the research institution, as well as by the Research Ethics Committee of the State University of Campinas.

### Healthy Work Environment Assessment Tool

HWEAT is a tool made up of 18 items distributed across six standard dimensions: Communication skills (items 1, 6 and 14), True collaboration (items 2, 10 and 15), Effectiveness in decision making (items 7, 11 and 16), Appropriate team (items 3, 8 and 12), Meaningful recognition (items 4, 9 and 17) and Authentic leadership (items 5, 13 and 18)^(7)^.

To evaluate each of these items, the participant indicates whether or not they agree with each of the statements, using a five-point Likert scale, which varies between one (totally disagree) and five (totally agree). From this, the higher the score, the greater the professionals’ agreement regarding the presence of standards in their work environment^(7)^.

The score is calculated by averaging the responses for each of the six domains, and for the total number of items, so that the scores obtained can be classified as excellent (4.00 - 5.00), good (3.00 - 3 .99) or require improvement (1.00 - 2.99). In this way, the tool identifies areas of improvement in each of the standards required for a healthy work environment. It is recommended that the target standard of results with the application of the tool is at least “good” for each of the standards, as well as for the total score^(7)^.

### Study design, period and location

Methodological study that followed six stages: translation, synthesis, back-translation, content validation by a group of experts, pre-test and evaluation of the process by the author of the original instrument^(8)^. The study was carried out between September 2022 and May 2023 and data collection took place in a hybrid manner, that is, online (committee of experts) and in person (pre-test). The fifth stage - pre-test, was carried out in a large public hospital, a reference for more than 40 municipalities, located in the interior of São Paulo, which provides tertiary and quaternary care. To describe the research, the criteria from the Consensus-Based Standards for the selection of health Measurement Instruments (COSMIN) checklist were adopted^(9)^.

### Sample and inclusion and exclusion criteria

As a sample size for the committee, a minimum of five experts^(10)^ who met the following inclusion criteria were considered: professionals from clinical practice or teaching, English teachers, experts in translation/validation studies or in the construct surrounding the instrument , and who, cumulatively, had at least four years of experience^(10-11)^. To classify specialists as junior, master or senior, the criteria described by Guimarães (2016)^(11)^ were used and adapted for the present study.

To select these experts, a search was carried out on the *Lattes* ^®^ Platform, of the National Council for Scientific and Technological Development, which took place in the form of an “advanced search”, in which the descriptors “Environment of Health Institutions” and “Studies of Validation”. In addition, the following filters were inserted: doctors and other researchers, Brazilians, who had updated their CVs in the last 24 months. On the date the search was carried out (20/10/2022), the result was a group of 29 researchers able to form the committee, however, one was excluded because she was one of the researchers in the present study.

From there, the 28 researchers were randomized by a statistical professional and the first 12 were invited to participate in the research, via email. The e-mails of these researchers were identified in the body of their CVs or in publications of articles they authored.

For the pre-test, whose objective is to evaluate whether the items are understandable by the target population, as well as the practical aspects of the application (administration time and usability)^(8,12)^, a minimum of 30 health professionals was considered. The sample for this stage was selected for convenience, considering as inclusion criteria doctors, nurses, nursing and pharmacy technicians, physiotherapists, pharmacists, speech therapists and nutritionists, who held positions linked to care or management and who worked in the institution. for at least three months. Those who left more than one item on the instrument blank were excluded.

The professionals were approached in their work units, explained about the research objectives and ethical aspects, and, after signing the TCLE, the researchers waited for the instruments to be completed by those who agreed to participate in the study.

### Study protocol

The cross-cultural adaptation process of the tool occurred in six stages. In the first, the HWEAT items were translated into Portuguese by two independent translators who were fluent in the original language of the instrument (English) and the target language (Portuguese) as their mother tongue. Thus, two translations were obtained, called T1 and T2. In the second stage, based on the versions produced, a third translator worked to resolve the word ambiguities between the translations, in order to reach a consensus and, in this way, the synthesis version was obtained (T12)^(8)^.

In possession of this version (T12), with the aim of verifying the existence of discrepancies in the meaning and content between the original and translated versions, the instrument was back-translated into the original language (English), by two translators who were not familiar with the original instrument and whose mother tongue was English, but were fluent in Portuguese. This step resulted in two back-translations, entitled BT1 and BT2^(8)^.

With all previously produced versions, in the fourth stage a form was created, using the Google Forms^®^ tool, to evaluate the content validity of the HWEAT synthesis version. After sending an invitation email, to those who accepted, the form link was sent, the first page of which contained the Free and Informed Consent Form. By clicking on “I accept to participate”, the participant was directed to complete the instruments: a characterization form in which sociodemographic and professional information was requested (gender, age, professional training, level of training, unit of activity, experience with studies of adaptation and validation of instruments and/or with the theme of work environment) and then questions related to the evaluation of semantic (meaning of words), idiomatic (referring to colloquial expressions), cultural (correspondence of terms of original version with the experiences of the target population) and conceptual (when the items actually evaluate the focus phenomenon)^(8)^, as well as clarity and relevance. For each of these assessments, a four-point Likert scale was used for each item.

Therefore, in relation to equivalences, experts could select the following options: 1) Not equivalent; 2) Requires major revision to be equivalent; 3) Requires minor revision to be equivalent or 4) Equivalent. Regarding clarity: 1) Not clear; 2) Unclear; 3) Clear or 4) Extremely clear^(10)^. And with regard to relevance: 1) Irrelevant; 2) Not very relevant; 3) Relevant or 4) Extremely relevant^(12)^.

For each item, the specialist who had selected answer options 1 or 2, for any of the evaluated criteria, was asked to leave contributions to improve the item. Based on the professionals’ response, a database was created and a quantitative evaluation stage was initiated, involving the calculation of the Content Validity Index (CVI), the Modified Kappa and the Content Validity Ratio (CVR). For the items in which the established minimum scores were not achieved, a qualitative stage was initiated and the suggestions made by the experts were analyzed, accepted and a new round of evaluation was started^(13)^.

After this stage, pre-testing began, at which point the tool was tested with professionals from the multidisciplinary health team. The purpose of this stage was to evaluate the practical aspects of applying the tool (administration time and usability)^(8,12)^. To do this, participants were asked to record the start and end time of filling out the instrument and at the end, answer three questions: 1) “Was it easy to understand the instructions for filling out the instrument?”; 2) “Was it easy to understand the items on the instrument?” and 3) “Was it easy to understand and mark the answers on the instrument?”. For each of these questions, a five-point Likert scale was used: 1) Totally Disagree, 2) Partially Disagree, 3) I have no opinion about, 4) Partially Agree and 5) Totally Agree^(11)^. In addition, contributions were requested from those who assigned a score of 1 or 2 to the questions mentioned above.

In the sixth and final stage, the transcultural adaptation process carried out was reported to the American Association of Critical-Care Nurses, which approved and authorized the publication of the results^(8)^.

### Results analysis

The data obtained during the research were tabulated in spreadsheets in Microsoft Excel for Windows^®^. Descriptive statistics of the qualitative variables and position measurements (mean, standard deviation, minimum, maximum) of the quantitative variables were performed.

Equivalences (semantic, idiomatic, cultural and conceptual) and clarity were analyzed by calculating the CVI - which represents the proportion of experts who agreed with the evaluated aspects and Modified Kappa - which assesses the chance of agreement between experts, in values ≥ 0.80 and ≥ 0.74, respectively, were considered satisfactory^(10)^. To analyze relevance, the CVR was calculated, using values ≥ 0.78 as a reference^(12)^. These analyzes were carried out using Statistical Analysis Software^®^ (SAS), version 9.4, by a statistical professional.

## RESULTS

The first three stages of the study were carried out by contracted companies, following the recommendations of the adopted framework and under the supervision of the researchers. In stage 4, the expert committee was made up of seven participants, six of whom were selected probabilistically from the *Lattes curriculum* (five nurses and one doctor) and one English teacher was selected for convenience.

The average age of the participants was 36.87 years (SD 13.52, Min: 33, Max: 72). Of these, six (85.71%) had a doctorate, four (57.14%) worked in teaching and research, two (28.57%) only in teaching and one (14.28%) specifically in research. The average experience in the profession was 19.85 years (SD 13.77, Min: 6, Max: 46) and all participants had experience with validation studies. According to the criteria adopted to classify the experts, four (57.1%) were seniors and three (42.9%) had masters.

Among the items evaluated, only two (items 5 and 13) did not reach the pre-established values for CVI and Modified Kappa, as can be seen in Table 1 and, therefore, were modified according to the suggestions received from experts. (deletion of the description “advanced practice nurses”) and forwarded to a second round of assessment.

**Table 1:** Content Validity Index, Modified Kappa and Content Validity Ratio of instrument items for equivalences, clarity and relevance, Campinas, São Paulo, Brazil, 2023

Items	Semantic Equivalence		Idiomatic Equivalence		Cultural Equivalence		Conceptual Equivalence		Clarity		Relevance
	CVI	Kappa	CVI	Kappa	CVI	Kappa	CVI	Kappa	CVI	Kappa	CVR
1	1.00	1.00	1.00	1.00	1.00	1.00	1.00	1.00	1.00	1.00	1.00
2	1.00	1.00	1.00	1.00	1.00	1.00	1.00	1.00	1.00	1.00	1.00
3	1.00	1.00	1.00	1.00	1.00	1.00	1.00	1.00	0.86	0.85	0.86
4	1.00	1.00	1.00	1.00	1.00	1.00	1.00	1.00	1.00	1.00	1.00
5	0.86	0.85	0.86	0.85	**0.71**	**0.66**	**0.71**	**0.66**	1.00	1.00	1.00
6	1.00	1.00	1.00	1.00	1.00	1.00	1.00	1.00	1.00	1.00	1.00
7	0.86	0.85	0.86	0.85	0.86	0.85	0.86	0.85	0.86	0.85	1.00
8	1.00	1.00	1.00	1.00	1.00	1.00	1.00	1.00	1.00	1.00	1.00
9	1.00	1.00	1.00	1.00	1.00	1.00	1.00	1.00	1.00	1.00	1.00
10	1.00	1.00	1.00	1.00	1.00	1.00	1.00	1.00	1.00	1.00	1.00
11	1.00	1.00	1.00	1.00	1.00	1.00	0.86	0.85	1.00	1.00	1.00
12	0.86	0.85	0.86	0.85	0.86	0.85	0.86	0.85	1.00	1.00	1.00
13	**0.71**	**0.66**	**0.71**	**0.66**	0.86	0.85	**0.71**	**0.66**	0.86	0.85	1.00
14	1.00	1.00	1.00	1.00	1.00	1.00	1.00	1.00	1.00	1.00	1.00
15	1.00	1.00	1.00	1.00	1.00	1.00	1.00	1.00	1.00	1.00	1.00
16	1.00	1.00	1.00	1.00	1.00	1.00	1.00	1.00	1.00	1.00	1.00
17	1.00	1.00	1.00	1.00	1.00	1.00	1.00	1.00	1.00	1.00	1.00
18	0.86	0.85	0.86	0.85	0.86	0.85	0.86	0.85	1.00	1.00	1.00

*CVI - Content Validity Index; CVR - Content Validity Ratio.*

In addition to these two items, the title of subscale 1 reached a CVI of 0.71 and a Modified Kappa of 0.66 and, therefore, was also changed from “Specialized communication” to “Communication skills”, as suggested by the experts and forwarded to the second round of evaluation. It is worth highlighting that item 18, despite having achieved satisfactory values in all criteria, also made reference to “advanced practice nurses”, as did items 5 and 13 and, therefore, this description was excluded by the researchers and the item forwarded to the second round of evaluation, in order to verify the experts’ agreement with such modification.

With regard to the evaluations of the instrument title, title of the other subscales, filling instructions and response scale, the values achieved in the tests were considered satisfactory, as they reached the established minimum values.

In the second round of evaluation, the CVI and Modified Kappa for the title of subscale 1 and for items 5 and 13 were 1.0 and 100% of experts agreed with the exclusion of the description “advanced practice nurses” from item 18.

At the end of content validation, the pre-test began with 31 healthcare professionals, whose average age was 36.87 years (SD 8.78, Min: 24, Max: 57) and average working time at the institution of 6.12 years (SD 7.97, Min: 0.33, Max: 30). Other aspects of the sample were presented in Table 2.

**Table 2:** Socio-professional variables of the sample participating in the pre-test, Campinas, São Paulo, Brazil, 2023

Variables	n	%
Gender		
Female	21	67.74
Male	10	32.26
Educational background		
High school and specific course	9	29.03
Undergraduate course	2	6.46
Post Graduation	6	19.35
Residency	4	12.90
Master’s degree	7	22.58
PhD	2	6.45
Others	1	3.23
Position		
Nursing assistant	8	25.81
Physician	8	25.81
Nurse	6	19.34
Physiotherapist	4	12.90
Farmacy technician	2	6.45
Outros	3	9.69
Sector		
Referenced Emergency Unit	14	45.16
Adult Inpatient Unit	10	32.26
Others (Pediatrics, outpatient clinics)	7	22.58

Regarding the practicality of applying the tool, the average completion time was 8.53 minutes (SD 7.45, Min: 3.0, Max: 36), and the majority of participants agreed that the instructions were easy to understand. filling (n=30; 96.77%), items (n=29; 93.55%) and answer options (n=30; 96.77%). The adapted scale is available online through a research data repository^(14)^.

## DISCUSSION

The objectives of this study were to adapt the HWEAT to Brazilian culture, evaluate the content validity of the instrument, as well as the practical aspects of its application. To adapt an instrument, it is necessary to use a methodical process, with a series of requirements, with the aim of achieving equivalence between the original and adapted versions^(8)^, a process that was systematically followed in the present study, thus as can be observed in other studies with similar objectives^(15-16)^.

Some authors consider that among the stages followed in this research, back translation could have been omitted, as there is no clear evidence to indicate that back translations improve the quality of the process and, therefore, costs and costs could be reduced. the time spent adapting instruments^(17)^. However, it was decided to maintain the back-translation stage in the present study, considering the methodological framework adopted, which recommends carrying out such a stage^(8)^.

In relation to the fourth stage of the study, the recommendations in the literature for the composition of the committee of experts were considered, regarding the number and characteristics of participants^(10-11)^, which was also verified in other studies^(15-16,18)^. However, it is noteworthy that, among these, only one evaluated^(14)^, in addition to equivalences, the clarity and relevance of the items.

In the present study, the majority of participants in the expert committee had a doctorate, worked in teaching and research in the area and had extensive experience in their professions. These characteristics made it possible to classify the majority as senior^(10)^, which contributed to the quality of the process conducted and brought evidence of content validity to the Brazilian version of the tool. Compared to other recent studies on cross-cultural adaptation and validation^(15-16,18-20)^, a gap was observed in the use of these criteria to classify experts.

It is also worth highlighting the fact that this stage was carried out online, which allowed us to overcome geographical limits and resources for face-to-face meetings. In this type of assessment, participants have the freedom to express themselves more freely, which contributes to greater neutrality in the process, due to the non-influence of the researchers and reinforces the internal reliability of the study^(21)^. Furthermore, another notable aspect of the present study was the fact that the selection of participants in the fourth stage occurred in a probabilistic manner, considered to be of greater statistical rigor. In another study that followed a similar theoretical methodological framework for adaptation, experts were purposefully recruited^(16)^.

Specifically analyzing the changes suggested by the experts, it was decided to exclude the description “advanced practice nurses”, as it was considered that Advanced Nursing Practice is not yet an established reality in the country. However, this practice has been researched and is considered promising as a new model of nursing care to be implemented, with pilot projects being developed in the country^(22)^. Therefore, it is possible that in the future the content of the Brazilian version of HWEAT will need to be reevaluated, if this practice becomes a reality in Brazil.

Furthermore, there were no indications from the pre-test participants to change the items due to lack of clarity or difficulty in understanding. The applicability time identified was relatively short, which may positively interfere with adherence and quality of responses from future participants^(23-24)^. The agreement above 93% regarding ease of understanding supports the use of the tool as a way of identifying opportunities for improvement and implementing strategies that contribute to healthier work environments.

It is noteworthy that HWEAT was also cross-culturally adapted for the Japanese^(20)^ and Canadian^(17)^ population and, for this culture, the cross-cultural adaptation process followed the same methodological framework as that of this study^(8)^. The global interest in research on the topic is in line with the Sustainable Development Goals, established by the United Nations, in which, more specifically in its eighth objective, it describes the importance of decent work for the economic growth of nations, whose nurses and the healthcare team has opportunities to contribute in a more significant way^(25)^. In this sense, the promotion of healthy and safe working environments is essential, as a way of protecting workers’ rights^(26)^.

### Study limitations

As a limitation of this study, we can mention the fact that the pre-test stage was carried out in a specific population scenario, restricting the cultural variability present in a country as large as Brazil. Furthermore, it can be considered the fact that the tool only had its content validity analyzed. Despite being essential, authors recommend that other measurement properties also be analyzed, before the instrument can actually be incorporated into practice^(8)^.

### Contributions to nursing

With regard to research, this study contributes to the development of new studies, firstly, to test the measurement properties of the tool such as construct validity and reliability and, secondly, to map and classify the work environments of health professionals.

With the availability of data collected using the Brazilian version of HWEAT, managers and researchers will be able to map the characteristics of environments to guide the establishment of priorities for the implementation of improvement actions, in addition to continuous monitoring of the relationship between the characteristics of the environment and the indicators performance related to patients, professionals and institutions.

Furthermore, the theme and the tool can also be presented to students in the health area as a way of awakening them to the need to reflect on the importance of implementing policies that guarantee appropriate teams, in addition to respect and development of leadership, communication, true collaboration, assertiveness in decision-making and significant recognition as a way of guaranteeing the well-being of workers who play essential roles in society.

## CONCLUSIONS

The HWEAT adaptation process for Brazilian culture was carefully followed as recommended by international literature, demonstrated evidence of content validity, as well as being a clear and easy-to-use tool.
