# Supplementary material for: Cross-cultural adaptation and validation of the Healthy Work Environment Assessment Tool in Brazilian culture
Source: Rev Bras Enferm. 2024 Sep 20;77(4):e20230505. doi: 10.1590/0034-7167-2023-0505 (PMC11419688; doi:10.1590/0034-7167-2023-0505)
Supplement: Translation

# Translation: Adaptação transcultural e validação *da Healthy Work Environment Assessment Tool* à cultura brasileira

**DOI:** 10.1590/0034-7167-2023-0505pt

**Authors:** Renata Cristina Gasparino, Sharlla Milênia Nogueira da Silva, Letícia Bianchini de Barros Minatogawa, Olga Maria Pimenta Lopes Ribeiro, Andrea Bernardes

**Affiliations:** I Universidade de São Paulo. Ribeirão Preto, São Paulo, Brasil; II Universidade Estadual de Campinas. Campinas, São Paulo, Brasil; III Escola Superior de Enfermagem do Porto. Porto, Portugal

**Keywords:** Tradução, Comparação Transcultural, Estudos de Validação, Ambiente de Instituições de Saúde, Condições de Trabalho

## Abstract

**Objetivos:**

adaptar e validar o conteúdo da *Healthy Work Environment Assessment Too*l para a cultura brasileira, e avaliar os aspectos práticos de sua aplicação.

**Métodos:**

estudo metodológico que seguiu seis estágios: tradução, síntese, retrotradução, validação de conteúdo por um grupo de especialistas, pré-teste e aprovação do processo pelo autor do instrumento original.

**Resultados:**

os três primeiros estágios foram realizados por empresas contratadas. No comitê, dois itens e o título de uma subescala foram avaliados em uma segunda rodada, na qual se alcançou um consenso entre os especialistas. No pré-teste, mais de 93% dos profissionais concordaram com a facilidade de compreensão da ferramenta. O tempo médio de preenchimento foi de 8,53 minutos. A *American Association of Critical-Care Nurses* autorizou a publicação dos resultados.

**Conclusões:**

a adaptação da ferramenta para a cultura brasileira foi concluída seguindo o referencial adotado. Além da evidência de validade de conteúdo, a ferramenta revela-se promissora para utilização gerencial.

## INTRODUÇÃO

Atualmente, tem-se avaliado a relação entre as características do ambiente de trabalho com a saúde dos profissionais, pois são vários os fatores que podem contribuir para implicações psicossociais e econômicas, não só no âmbito individual, como também familiar, organizacional e social. Dessa forma, nota-se o ambiente de trabalho como uma crucial “arena” para promoção da saúde da população, que não pode ser minimizada^(1)^.

Nesse sentido, a Organização Mundial da Saúde (OMS) definiu um ambiente saudável de trabalho como um local em que “os trabalhadores e gestores colaboram para o uso de um processo de melhoria contínua da proteção e promoção da segurança, saúde e bem-estar de todos os trabalhadores e para a sustentabilidade do ambiente de trabalho”^(2)^. Esse construto é considerado fundamental na área da saúde, sobretudo para garantir atenção à saúde dos envolvidos; satisfação e retenção dos profissionais; segurança do paciente; além de colaborar para manter a viabilidade financeira das organizações^(3-4)^.

Ademais, o tema ambiente saudável de trabalho foi foco do 11º Seminário Institucional do Conselho Federal de Enfermagem (Cofen), realizado em 2021. No evento, foi considerado que o Brasil vive uma segunda pandemia em consequência da Covid-19, agora relacionada à saúde mental^(5)^. Essa avaliação foi reforçada com o relatório da Organização Internacional do Trabalho (OIT) que destaca que, apesar do trabalho atuar como um potencial fator protetor da saúde mental ao proporcionar estrutura, interações sociais, senso de esforço e propósito coletivos, também pode contribuir para um processo de adoecimento psíquico. Com isso, estima-se que 12 bilhões de dias de trabalho são perdidos anualmente por causa de depressão e ansiedade^(6)^.

Para desenvolver um ambiente saudável de trabalho, a OMS indica a necessidade em se realizar um diagnóstico inicial, para posteriormente, os gestores poderem desenvolver um processo de melhoria contínua. As etapas iniciais deste processo consistem na mobilização de trabalhadores e empregadores, bem como reuniões de equipes de trabalho para promover mudanças no ambiente^(2)^. Dessa forma, importa destacar que a utilização de ferramentas com evidências de validade e confiabilidade são fundamentais para avaliar determinados padrões presentes nos ambientes e guiar transformações nos locais nos quais o trabalho é desenvolvido.

Em vista disso, a *American Association of Critical-Care Nurses* (AACN) desenvolveu a *Healthy Work Environment Assessment Tool* (HWEAT), um instrumento que tem por finalidade servir como facilitador na identificação de focos de melhoria no ambiente de trabalho na área da saúde. Além disso, destaca-se por sua capacidade de ser aplicada em diferentes unidades e em considerar a percepção de toda a equipe multiprofissional^(7)^, o que potenciará a possibilidade de gerar mudanças que impactem positivamente na qualificação dos ambientes de trabalho e, simultaneamente, na saúde e bem-estar de toda a equipe. Acresce que a HWEAT foi considerada, por autores que conduziram uma revisão sistemática, como um dos três instrumentos mais utilizados para se avaliar as características do ambiente de trabalho^(3)^.

Essa ferramenta teve seus itens divididos em seis padrões: comunicação, colaboração verdadeira, efetividade na tomada de decisões, equipe apropriada, reconhecimento significativo e liderança autêntica. Vale ressaltar que os padrões conduzem a melhoria contínua da qualidade^(7)^. Além disso, autores evidenciaram que o ambiente de trabalho com características favoráveis é um fator central de impacto para se obter resultados satisfatórios relacionados a enfermeiros, pacientes e instituições^(3)^. A HWEAT teve sua confiabilidade testada junto à equipe multiprofissional e alcançou valores de alfa de Cronbach que variaram entre entre 0,77 - 0,81^(7)^.

Considerando que a literatura brasileira ainda não dispõe de um instrumento que avalie as características de um ambiente saudável de trabalho na perspectiva da equipe multiprofissional, e que há evidências que apontam para a necessidade da realização de avaliações dos ambientes de trabalho na área da saúde, acredita-se que a disponibilização da HWEAT para a cultura brasileira poderá contribuir para a identificação de focos de melhoria e de desenvolvimento de estratégias que poderão contribuir para a promoção de ambientes mais saudáveis. E, consequentemente, para o alcance de resultados mais favoráveis para os profissionais, os pacientes e as instituições.

## OBJETIVOS

Adaptar e validar o conteúdo da *Healthy Work Environment Assessment Tool* para a cultura brasileira, além de avaliar os aspectos práticos de sua aplicação.

## MÉTODOS

### Aspectos éticos

O autor do instrumento original concedeu anuência para o processo de adaptação transcultural e validação da HWEAT para o contexto brasileiro. Além disso, o estudo foi aprovado pelos responsáveis da instituição sede da pesquisa, bem como pelo Comitê de Ética em Pesquisa da Universidade Estadual de Campinas.

### Healthy Work Environment Assessment Tool

A HWEAT é uma ferramenta composta por 18 itens distribuídos em seis padrões: Habilidades de comunicação (itens 1, 6 e 14), Colaboração verdadeira (itens 2, 10 e 15), Efetividade na tomada de decisões (itens 7, 11 e 16), Equipe apropriada (itens 3, 8 e 12), Reconhecimento significativo (itens 4, 9 e 17) e Liderança autêntica (itens 5, 13 e 18)^(7)^.

Para avaliar cada um desses itens, o participante assinala se concorda ou não com cada uma das afirmações, por meio de uma escala Likert com cinco pontos, que varia entre um (discordo totalmente) e cinco (concordo totalmente). A partir disso, quanto maior o escore, maior a concordância dos profissionais em relação à presença dos padrões no seu ambiente de trabalho^(7)^.

Consequentemente, o escore é calculado pela média das respostas para cada um dos seis domínios e, também, para o total de itens, de modo que os escores obtidos podem ser classificados em excelentes (4,00 - 5,00), bons (3,00 - 3,99) ou necessitam de melhorias (1,00 - 2,99). Dessa forma, a ferramenta identifica focos de melhoria em cada um dos padrões requeridos para um ambiente saudável de trabalho. Além do mais, recomenda-se que o padrão alvo dos resultados com a aplicação da ferramenta seja no mínimo “bom” para cada um dos padrões, bem como para o escore total^(7)^.

### Desenho, período e local do estudo

Estudo metodológico que seguiu seis estágios: tradução, síntese, retrotradução, validação de conteúdo por um grupo de especialistas, pré-teste e avaliação do processo pelo autor do instrumento original^(8)^. O estudo foi realizado entre os meses de setembro de 2022 a maio de 2023 e a coleta de dados ocorreu de forma híbrida, ou seja, de maneira on-line (comitê de especialistas) e presencial (pré-teste). O quinto estágio, ou seja, o pré-teste, foi realizado em um hospital público, de grande porte e referência para mais de 40 municípios, localizado no interior de São Paulo, que realiza atendimentos de nível terciário e quaternário. Para a descrição da pesquisa, foram adotados os critérios do checklist *Consensus-Based Standards for the selection of health Measurement Instruments* (COSMIN)^(9)^.

### Amostra e critérios de inclusão e exclusão

Como tamanho amostral para o comitê, foi considerado um mínimo de cinco especialistas^(10)^ que atendessem aos seguintes critérios de inclusão: profissionais da prática clínica ou docência; professores de inglês; experts em estudos de tradução/validação ou no construto que envolve o instrumento, e que, cumulativamente, apresentassem no mínimo, quatro anos de experiência^(10-11)^. Para classificar os especialistas em júnior, master ou sênior, os critérios descritos por Guimarães (2016)^(11)^ foi utilizado e adaptado para o presente estudo.

Logo, para a seleção desses especialistas foi realizada uma busca na Plataforma *Lattes^®^*, do Conselho Nacional de Desenvolvimento Científico e Tecnológico, que ocorreu na forma de “busca avançada”, em que foram utilizados os descritores “Ambiente de Instituições de Saúde” e “Estudos de Validação”. Além disso, foram inseridos os seguintes filtros: doutores e demais pesquisadores, brasileiros e que tivessem atualizado seus currículos nos últimos 24 meses. Na data em que a busca foi realizada (20/10/2022), foi obtido como resultado um grupo de 29 pesquisadores aptos a compor o comitê, entretanto, um foi excluído por se tratar de uma das pesquisadoras do presente estudo.

A partir disso, os 28 pesquisadores foram aleatorizados por um profissional estatístico e os primeiros 12 foram convidados a participar da pesquisa, por meio de correio eletrônico. Os *e-mails* destes pesquisadores foram identificados no corpo de seus currículos ou em publicações de artigos de sua autoria.

Já para o pré-teste, cujo objetivo é avaliar se os itens são compreensíveis pela população alvo, assim como os aspectos práticos da aplicação (tempo de administração e usabilidade)^(8,12)^, foi considerado um mínimo de 30 profissionais de saúde. A amostra para essa etapa foi selecionada por conveniência, sendo considerados como critérios de inclusão profissionais médicos, enfermeiros, técnicos de enfermagem e de farmácia, fisioterapeutas, farmacêuticos, fonoaudiólogos e nutricionistas, que exercessem cargos ligados à assistência ou à gestão e que atuassem na instituição por, pelo menos, três meses. Foram excluídos aqueles que deixaram mais do que um item do instrumento em branco. Além disso, os profissionais foram abordados em suas unidades de trabalho, esclarecidos sobre os objetivos da pesquisa e aspectos éticos, e, após assinatura do TCLE, as pesquisadoras aguardaram o preenchimento dos instrumentos daqueles que aceitaram participar do estudo.

### Protocolo do estudo

O processo de adaptação transcultural da ferramenta ocorreu em seis estágios. No primeiro, os itens da HWEAT foram traduzidos para o português por dois tradutores independentes, que possuíam fluência no idioma original do instrumento (inglês) e a língua alvo (português) como materna. Assim, obteve-se duas traduções, denominadas T1 e T2. No segundo estágio, a partir das versões produzidas, um terceiro tradutor atuou para que as ambiguidades de palavras entre as traduções fossem solucionadas, de forma a obter um consenso e, dessa forma, obteve-se a versão síntese(T12)^(8)^.

Em posse desta versão (T12), com o objetivo de verificar a existência de discrepâncias no significado e conteúdo entre as versões original e traduzida, o instrumento foi retrotraduzido para o idioma original (inglês), por dois tradutores que não conheciam o instrumento original, cuja língua materna era o inglês, mas tinham fluência em português. Esta etapa resultou em duas retrotraduções, intituladas BT1 e BT2^(8)^.

Com todas as versões produzidas anteriormente, no quarto estágio foi elaborado um formulário, por meio da ferramenta Google Formulários^®^, para avaliação da validade de conteúdo da versão síntese da HWEAT. Após o envio de um e-mail convite, para aqueles que aceitaram, foi enviado o link do formulário, cuja primeira página continha o Termo de Consentimento Livre e Esclarecido (TCLE). Ao clicar em “Aceito participar”, o participante era direcionado para o preenchimento dos instrumentos: uma ficha de caracterização em que foram solicitadas informações sociodemográficas e profissionais (sexo, idade, formação profissional, nível de formação, unidade de atuação, experiência com estudos de adaptação e validação de instrumentos e/ou com o tema ambiente de trabalho). Em seguida, foram apresentadas as questões relacionadas à avaliação das equivalências semântica (significado das palavras), idiomática (referente a expressões coloquiais), cultural (correspondência dos termos da versão original com as experiências da população-alvo) e conceitual (quando os itens de fato avaliam o fenômeno foco)^(8)^, bem como da clareza e da relevância. Para cada uma dessas avaliações foi utilizada, para cada item, uma escala tipo Likert com quatro pontos.

Dessa forma, em relação às equivalências, os especialistas poderiam assinalar as seguintes opções: 1) Não equivalente; 2) Necessita de grande revisão para ser equivalente; 3) Necessita de pequena revisão para ser equivalente; ou 4) Equivalente. Quanto à clareza: 1) Não claro; 2) Pouco claro; 3) Claro; ou 4) Extremamente claro^(10)^. E no que diz respeito à relevância: 1) Irrelevante; 2) Pouco relevante; 3) Relevante; ou 4) Extremamente relevante^(12)^.

Com efeito, para cada item foi solicitado ao especialista que havia assinalado as opções de resposta 1 ou 2, para qualquer um dos critérios avaliados, que deixasse contribuições para melhoria do item. A partir da resposta dos profissionais, um banco de dados foi elaborado e uma etapa de avaliação quantitativa foi iniciada, envolvendo o cálculo do Índice de Validade de Conteúdo (IVC), do *Kappa* Modificado e da *Content Validity Ratio* (CVR). Para os itens em que não se alcançou os escores mínimos estabelecidos, uma etapa qualitativa foi iniciada e as sugestões feitas pelos especialistas foram analisadas, acatadas e uma nova rodada de avaliação foi iniciada^(13)^.

Findado este estágio, deu-se início ao pré-teste, momento no qual a ferramenta foi testada junto aos profissionais da equipe multidisciplinar de saúde. A finalidade desta etapa foi avaliar os aspectos práticos da aplicação da ferramenta (tempo de administração e usabilidade)^(8,12)^. Para isso, foi solicitado aos participantes que registrassem o horário de início e término de preenchimento do instrumento, e ao final respondessem três perguntas: 1) “Foi fácil compreender as instruções de preenchimento do instrumento?”; 2) “Foi fácil compreender os itens do instrumento?”; e 3) “Foi fácil compreender e assinalar as respostas do instrumento?”. Para cada uma dessas perguntas foi utilizada uma escala Likert com cinco pontos sendo: 1) Discordo Totalmente, 2) Discordo Parcialmente, 3) Não tenho opinião sobre, 4) Concordo Parcialmente e 5) Concordo Totalmente^(11)^. Além disso, foi solicitada a contribuição com sugestões para aqueles que atribuíssem pontuação 1 ou 2 às perguntas citadas acima.

Por fim, no sexto e último estágio, o processo de adaptação transcultural realizado foi reportado à *American Association of Critical-Care Nurses* que aprovou e autorizou a publicação dos resultados^(8)^.

### Análise dos resultados

Os dados obtidos durante a pesquisa foram tabulados em planilhas no *Microsoft Excel for Windows* ^®^. Além disso, foram realizadas estatísticas descritivas das variáveis qualitativas e medidas de posição (média, desvio-padrão, mínimo, máximo) das variáveis quantitativas.

As equivalências (semântica, idiomática, cultural e conceitual) e clareza foram analisadas por meio do cálculo do IVC - que representa a proporção de especialistas que concordaram com os aspectos avaliados e do Kappa Modificado - que avalia a chance de concordância entre os especialistas; em que ambos foram considerados satisfatórios valores ≥ 0,80 e ≥ 0,74, respectivamente^(10)^. Para análise da relevância foi calculada a CVR, tendo como referência valores ≥ 0,78(12). Essas análises foram realizadas no software *Statistical Analysis Software* ^®^ (SAS), versão 9.4, por um profissional estatístico.

## RESULTADOS

Os três primeiros estágios do estudo foram realizados por empresas contratadas, seguindo as recomendações do referencial adotado e sob supervisão das pesquisadoras. No estágio 4, o comitê de especialistas foi composto por sete participantes, sendo que seis foram selecionados de forma probabilística, a partir do currículo *Lattes* (cinco enfermeiras e um médico), além do mais um professor de inglês foi selecionado por conveniência.

A idade média dos participantes foi de 36,87 anos (DP 13,52, Mín.: 33, Máx.: 72). Destes, seis (85,71%) possuíam doutorado, quatro (57,14%) trabalhavam com ensino e pesquisa, dois (28,57%) somente com ensino e um (14,28%), especificamente, com pesquisa. A média de experiência na profissão foi de 19,85 anos (DP 13,77, Mín.: 6, Máx.: 46) e todos os participantes possuíam experiência com estudos de validação. De acordo com os critérios adotados para classificação dos especialistas, quatro (57,1%) eram seniores e três (42,9%) eram mestres.

Dentre os itens avaliados, apenas dois (itens 5 e 13) não alcançaram os valores pré-estabelecidos para o IVC e o Kappa Modificado, como pode ser observado na Tabela 1 e, por isso, foram modificados de acordo com as sugestões recebidas dos especialistas (exclusão da descrição “enfermeiros de prática avançada”) e, depois, encaminhados para uma segunda rodada de avaliação.

**Tabela 1:** Índice de Validade de Conteúdo, Kappa Modificado e *Content Validity Ratio* dos itens do instrumento para as equivalências, clareza e relevância, Campinas, São Paulo, Brasil, 2023

Itens	Equivalência Semântica		Equivalência Idiomática		Equivalência Cultural		Equivalência Conceitual		Clareza		Relevância
	IVC	Kappa	IVC	Kappa	IVC	Kappa	IVC	Kappa	IVC	Kappa	CVR
1	1,00	1,00	1,00	1,00	1,00	1,00	1,00	1,00	1,00	1,00	1,00
2	1,00	1,00	1,00	1,00	1,00	1,00	1,00	1,00	1,00	1,00	1,00
3	1,00	1,00	1,00	1,00	1,00	1,00	1,00	1,00	0,86	0,85	0,86
4	1,00	1,00	1,00	1,00	1,00	1,00	1,00	1,00	1,00	1,00	1,00
5	0,86	0,85	0,86	0,85	**0,71**	**0,66**	**0,71**	**0,66**	1,00	1,00	1,00
6	1,00	1,00	1,00	1,00	1,00	1,00	1,00	1,00	1,00	1,00	1,00
7	0,86	0,85	0,86	0,85	0,86	0,85	0,86	0,85	0,86	0,85	1,00
8	1,00	1,00	1,00	1,00	1,00	1,00	1,00	1,00	1,00	1,00	1,00
9	1,00	1,00	1,00	1,00	1,00	1,00	1,00	1,00	1,00	1,00	1,00
10	1,00	1,00	1,00	1,00	1,00	1,00	1,00	1,00	1,00	1,00	1,00
11	1,00	1,00	1,00	1,00	1,00	1,00	0,86	0,85	1,00	1,00	1,00
12	0,86	0,85	0,86	0,85	0,86	0,85	0,86	0,85	1,00	1,00	1,00
13	**0,71**	**0,66**	**0,71**	**0,66**	0,86	0,85	**0,71**	**0,66**	0,86	0,85	1,00
14	1,00	1,00	1,00	1,00	1,00	1,00	1,00	1,00	1,00	1,00	1,00
15	1,00	1,00	1,00	1,00	1,00	1,00	1,00	1,00	1,00	1,00	1,00
16	1,00	1,00	1,00	1,00	1,00	1,00	1,00	1,00	1,00	1,00	1,00
17	1,00	1,00	1,00	1,00	1,00	1,00	1,00	1,00	1,00	1,00	1,00
18	0,86	0,85	0,86	0,85	0,86	0,85	0,86	0,85	1,00	1,00	1,00

*IVC - Índice de Validade de Conteúdo; CVR - Content Validity Ratio.*

Além desses dois itens, o título da subescala 1 alcançou um IVC de 0,71 e um Kappa Modificado de 0,66 e, por isso, também foi alterado de “Comunicação especializada” para “Habilidade de comunicação”, conforme sugestão dos especialistas, e encaminhado para a segunda rodada de avaliação. Vale destacar que o item 18, apesar de ter alcançado valores satisfatórios em todos os critérios, também fazia referência aos “enfermeiros de prática avançada”, assim como os itens 5 e 13 e, por conseguinte, essa descrição foi excluída pelas pesquisadoras e o item reencaminhado para a segunda rodada de avaliação, com o intuito de verificar a concordância dos especialistas com tal modificação.

No que se refere às avaliações do título do instrumento, título das demais subescalas, instruções de preenchimento e escala de respostas, os valores alcançados nos testes foram considerados satisfatórios, na medida em que atingiram os valores mínimos estabelecidos. Salienta-se ainda que na segunda rodada de avaliação, o IVC e o Kappa Modificado para o título da subescala 1 e para os itens 5 e 13 foram de 1,0 e 100% dos especialistas concordaram com a exclusão da descrição “enfermeiros de prática avançada” do item 18.

Ao final da validação de conteúdo, foi iniciado o pré-teste junto a 31 profissionais da área da saúde, cuja idade média foi de 36,87 anos (DP 8,78, Mín.: 24, Máx.: 57) e tempo médio de trabalho na instituição de 6,12 anos (DP 7,97, Mín.: 0,33, Máx.: 30). Já os demais aspectos da amostra foram apresentados na Tabela 2.

**Tabela 2:** Variáveis socioprofissionais da amostra participante do pré-teste, Campinas, São Paulo, Brasil, 2023

Variável	n	%
Gênero		
Feminino	21	67,74
Masculino	10	32,26
Nível de formação		
Técnico nível médio	9	29,03
Graduação	2	6,46
Especialização	6	19,35
Residência	4	12,90
Mestrado	7	22,58
Doutorado	2	6,45
Outros	1	3,23
Cargo		
Técnico em Enfermagem	8	25,81
Médico	8	25,81
Enfermeiro	6	19,34
Fisioterapeuta	4	12,90
Técnico em Farmácia	2	6,45
Outros	3	9,69
Setor		
Unidade de Emergência Referenciada	14	45,16
Unidade de Internação de Adultos	10	32,26
Outros (Pediatria, ambulatórios)	7	22,58

Em relação à praticabilidade da aplicação da ferramenta, o tempo médio de preenchimento foi de 8,53 minutos (DP 7,45, Mín.: 3,0, Máx.: 36), e a maioria dos participantes concordou com a facilidade da compreensão das instruções de preenchimento (n=30; 96,77%), dos itens (n=29; 93,55%) e das opções de resposta (n=30; 96,77%). A escala adaptada está disponível de maneira on-line através de repositório de dados de pesquisa^(14)^.

## DISCUSSÃO

Os objetivos deste estudo foram adaptar a HWEAT para a cultura brasileira, avaliar a validade de conteúdo do instrumento, bem como os aspectos práticos da sua aplicação. Para adaptar um instrumento, faz-se necessário o uso de um processo metódico, com uma série de exigências, com o intuito de atingir equivalência entre as versões original e a adaptada^(8)^. Este processo foi sistematicamente seguido no presente estudo, assim como pode ser observado em outros estudos com objetivos similares^(15-16)^.

Alguns autores consideram que dentre os estágios seguidos nesta pesquisa, a realização da retrotradução poderia ter sido omitida, na medida em que não existem evidências claras que indiquem que as *back translations* melhoram a qualidade do processo e, por isso, poderiam ser reduzidos os custos e o tempo empregados na adaptação de instrumentos^(17)^. Entretanto, optou-se por manter o estágio das retrotraduções no presente estudo, considerando o referencial metodológico adotado, que recomenda a realização de tal etapa^(8)^.

Em relação ao quarto estágio do estudo, foram contempladas as recomendações da literatura para composição do comitê de especialistas, no que concerne à quantidade e às características dos participantes^(10-11)^, o que também foi verificado em outros estudos^(15-16,18)^. Porém, dentre estes, destaca-se que somente um avaliou^(14)^ para além das equivalências, também a clareza e a relevância dos itens.

No presente estudo, a maioria dos participantes do comitê de especialistas possuía doutorado, trabalhava com ensino e pesquisa na área e possuía vasto tempo de experiência em suas profissões. Tais características permitiram classificar a maioria em sênior^(10)^, o que colaborou para a qualidade do processo conduzido e trouxe à versão brasileira da ferramenta evidências de validade de conteúdo. Comparativamente a outros estudos recentes de adaptação transcultural e validação^(15-16,18-20)^, os quais observou-se uma lacuna no uso destes critérios para classificação dos especialistas.

Ainda, destaca-se o fato desta etapa ter sido realizada de maneira on-line, o que propiciou a superação de limites geográficos e de recursos para reuniões presenciais. Neste tipo de avaliação, os participantes têm liberdade para se expressarem mais livremente, o que contribui para maior neutralidade do processo, em decorrência da não influência das pesquisadoras, e reforça a confiabilidade interna do estudo^(21)^. Ademais, outro aspecto de destaque no presente estudo, foi o fato da seleção dos participantes do quarto estágio ter ocorrido de maneira probabilística, considerada de maior rigor estatístico. Em outro estudo que seguiu um referencial teórico metodológico semelhante para a adaptação, os especialistas foram propositadamente recrutados^(16)^.

Analisando especificamente as alterações sugeridas pelos especialistas, foi decidido pela exclusão da descrição “enfermeiros de prática avançada”, pois considerou-se que a Prática Avançada de Enfermagem ainda não é uma realidade estabelecida no país. Entretanto, essa prática vem sendo pesquisada e é considerada promissora como um novo modelo de assistência de enfermagem a ser implementado, havendo projetos pilotos em desenvolvimento na nação^(22)^. Dessa forma, é possível que futuramente o conteúdo da versão brasileira da HWEAT necessite ser reavaliado, caso essa prática torne-se uma realidade no Brasil.

Ademais, não houve indicações por parte dos participantes do pré-teste para alterações dos itens por falta de clareza ou dificuldade de compreensão. O tempo de aplicabilidade identificado foi relativamente curto, o que pode interferir positivamente na adesão e na qualidade das respostas de futuros participantes^(23-24)^. A concordância acima de 93% em relação à facilidade de compreensão, corrobora para utilização da ferramenta como forma de identificar oportunidades de melhorias e implementação de estratégias que contribuam para ambientes mais saudáveis de trabalho.

Portanto, ressalta-se que a HWEAT também foi adaptada transculturalmente para a população japonesa^(20)^ e canadense^(17)^ e, para essas culturas, o processo de adaptação transcultural seguiu o mesmo referencial metodológico que o deste estudo^(8)^. O interesse global em pesquisas sobre o tema vai ao encontro dos Objetivos do Desenvolvimento Sustentável, estabelecidos pela Organização das Nações Unidas, em que, mais especificamente em seu oitavo objetivo, descreve a importância do trabalho digno para o crescimento econômico das nações, cujos enfermeiros e a equipe de saúde têm oportunidades de contribuir de forma mais significativa^(25)^. Nesse sentido, é primordial a promoção de ambientes de trabalho saudáveis e seguros, como forma de proteger os direitos dos trabalhadores^(26)^.

### Limitações do estudo

Enquanto limitação deste estudo, pode-se citar o fato da etapa de pré-teste ter sido realizada em um cenário populacional específico, restringindo a variabilidade cultural presente em um país tão extenso como o Brasil. Além disso, pode ser considerado o fato de a ferramenta ter apenas a sua validade de conteúdo analisada. Apesar de essencial, autores recomendam que outras propriedades de medida também sejam analisadas, antes do instrumento poder ser, de fato, incorporado à prática^(8)^.

### Contribuições para a área de enfermagem

No que se refere à pesquisa, este estudo contribui para que novos estudos sejam desenvolvidos. Primeiramente, para testar as propriedades de medida da ferramenta como validade de construto e confiabilidade e, em seguida, para mapear e classificar os ambientes de trabalho dos profissionais de saúde.

Outrossim, com a disponibilização dos dados coletados pelo uso da versão brasileira da HWEAT, gestores e pesquisadores poderão mapear as características dos ambientes para guiar o estabelecimento de prioridades de implementação de ações de melhoria, além do monitoramento contínuo da relação entre as características do ambiente e os indicadores de desempenho relacionados aos pacientes, profissionais e instituições.

Ademais, o tema e a ferramenta também poderão ser apresentados aos estudantes da área da saúde como forma de despertá-los para a necessidade de reflexão sobre a importância da implementação de políticas que garantam equipes apropriadas, além do respeito e do desenvolvimento das lideranças, da comunicação, da colaboração verdadeira, da assertividade na tomada de decisões e do reconhecimento significativo como forma de garantir o bem-estar de trabalhadores que exercem papéis essenciais perante a sociedade.

## CONCLUSÕES

Em suma, o processo de adaptação da HWEAT para a cultura brasileira foi criteriosamente seguido, conforme preconiza a literatura internacional. Além do mais, demonstrou evidências de validade de conteúdo, bem como ser uma ferramenta clara e de fácil utilização.
